# The Relationship Between Glycemic Variability and Inflammatory Markers in Obese Children with Insulin Resistance and Metabolic Syndrome

**DOI:** 10.4274/jcrpe.4031

**Published:** 2017-09-01

**Authors:** Abdurrahman Kaya, Cemil Koçyiğit, Gönül Çatlı, Elif Büşra Özkan, Bumin Nuri Dündar

**Affiliations:** 1 Tepecik Training and Research Hospital, Clinic of Pediatrics, İzmir, Turkey; 2 İzmir Katip Çelebi University Faculty of Medicine, Department of Pediatric Endocrinology, İzmir, Turkey; 3 İzmir Katip Çelebi University Faculty of Medicine, İzmir, Turkey

**Keywords:** Glycemic variability, metabolic syndrome, Interleukin-6, Adiponectin

## Abstract

**Objective:**

Increased glycemic variability (GV) is associated with increased oxidative stress, vascular complications, and mortality in metabolic syndrome (MS) and diabetes mellitus patients. To investigate the relationship between GV and inflammatory parameters in obese children with insulin resistance (IR) and to elucidate their effects on the development of MS.

**Methods:**

Fifty obese adolescents with IR were included in the study. All patients underwent anthropometric measurements, body fat analysis, and continuous glucose monitoring system (CGMS) for 24 hours. Serum lipids, adiponectin, and interleukin-6 (IL-6) levels were measured. GV coefficient (GVC) was calculated using the standard deviation and the average glucose value obtained by CGMS. IR was diagnosed according to the results of oral glucose tolerance test (OGTT). MS was diagnosed according to the modified World Health Organization and the International Diabetes Federation criteria.

**Results:**

Twenty-seven of the patients had MS and the remaining had only IR. Body fat mass, HbA1c, IL-6 levels, and peak insulin levels in the OGTT were significantly higher in the group with MS, but there was no difference in adiponectin levels. GVC was not different between the groups, but GVC significantly positively correlated with homeostasis model of assessment for IR, as well as with fasting, peak, and total insulin levels when all the patients were analyzed, while no significant relation was detected with adiponectin and IL-6 levels.

**Conclusion:**

This study suggests that GV is not different among obese adolescents with IR and MS. There seems to be a significant association between GV and IR parameters. However, other diagnostic criteria of MS (hypertension and/or dyslipidemia) or elevated IL-6 levels does not cause further increase in GV.

**What is already known on this topic?**

Glycemic variability describes the fluctuations in blood glucose and it is associated with vascular complications and mortality in patients with metabolic syndrome and diabetes mellitus.

**What this study adds?**

Glycemic variability is not different among obese adolescents with insulin resistance and metabolic syndrome. Elevated interleukin-6 levels and metabolic syndrome diagnostic criteria such as hypertension and dyslipidemia do not cause further increase in glycemic variability.

## INTRODUCTION

Glycemic variability (GV) describes the fluctuations in blood glucose levels throughout the day. Regardless of the average blood glucose concentration, increased GV has been shown to increase oxidative stress and cause endothelial dysfunction. It is also associated with vascular complications and mortality in patients with metabolic syndrome (MS) and diabetes mellitus (DM) (1). The fluctuations in blood glucose concentration have been reported to adversely affect endothelial function even in non-diabetic individuals (2). Furthermore, people who develop diabetes have an increased cardiovascular risk even before the appearance of diabetes and subjects with MS are at high risk for both cardiovascular events and diabetes (3). MS is associated with increased circulating levels of proinflammatory cytokines such as interleukin-6 (IL-6) and decreased anti-inflammatory factors such as adiponectin, which are both able to influence insulin sensitivity and endothelial dysfunction (4,5). Although there are studies investigating the relationship of GV with endothelial dysfunction and DM complications in adults with MS, studies in children are limited to type 1 diabetes. There are no reports investigating the relationship between GV and inflammatory markers in childhood obesity.

In this study, we aimed to investigate the relationship between GV by continuous glucose monitoring system (CGMS) and inflammatory parameters in obese children with insulin resistance (IR) and to elucidate their effects on the development of MS.

## METHODS

This study was conducted at İzmir Tepecik Training and Research Hospital in patients attending the Pediatric Endocrinology outpatient clinic between November 2014 and May 2015. The study protocol was approved by the Ethics Committee of İzmir Katip Çelebi University Faculty of Medicine. Written consent was obtained from all subjects and their parents before the study. The criteria defined by the World Health Organization and International Diabetes Federation were used in the diagnosis of MS (6,7). Patients who met both criteria were accepted as MS.

Fifty patients 10-18 years of age with obesity and IR were enrolled in the study. All subjects underwent a detailed physical examination including evaluation for syndromes and endocrine diseases as well as a laboratory evaluation including thyroid function tests and diurnal cortisol levels. Participants with syndromic obesity, endocrine disorders accompanied by obesity, a history of drug use (glucocorticoid, antipsychotics, etc.), and metabolic, cardiovascular, respiratory or hepatic disease were excluded.

Anthropometric measurements [height, body weight, waist circumference (WC)] were performed by the same person using the same tools and the results were recorded. The height and weight of each participant were measured while participants were wearing a light robe and no shoes. Subjects with a body mass index (BMI) value greater or equal to the 95^th^ percentile according to the age and gender were considered as obese. The BMI percentile and BMI standard deviation (SD) were evaluated using the reference values developed by Bundak et al (8). WC was measured at the narrowest point between the lower border of the rib cage and the iliac crest. WC was evaluated using the percentile curves of healthy Turkish children (9). BMI was calculated by dividing weight by height in meters squared (kg/m^2^). Blood pressure was measured twice from the right brachial artery in a sitting position following a 10-minute rest. The average of these two measurements was recorded. Children with systolic and/or diastolic blood pressure greater than the 95^th^ percentile (adjusted for height, age, and sex) were considered to have hypertension (10). Body composition of patients, i.e. body fat percentage (%), fat mass (FM), fat-free mass, and muscle mass, was analyzed using the bioelectrical impedance device (TBF-310GS^TM^, Tanita, Tokyo, Japonya).

Following overnight fasting, the blood samples were collected for estimation of biochemical parameters such as glucose level, lipid profile [triglyceride, low-density lipoprotein cholesterol (LDL-C), and high-density lipoprotein cholesterol levels] and insulin levels. An oral glucose tolerance test (OGTT) was performed in all patients. Blood glucose and insulin concentrations were measured before and at 30, 60, 90, and 120 minutes after the consumption of a glucose load in a dose of 1.75 g per kilogram of body weight (up to a maximum of 75 g of glucose). The homeostasis model of assessment for IR (HOMA-IR) index was implemented using the following equation: [fasting insulin (mU/L) x fasting glucose (mmol/L)/22.5]. Impaired fasting glucose (fasting glucose 100-125 mg/dL), impaired glucose tolerance (2-h glucose 140-199 mg/dL), and diabetes (fasting glucose ≥126 mg/dL or 2-h glucose ≥200 mg/dL) were defined by glucose levels obtained during the OGTT according to the American Diabetes Association guidelines (11). IR was considered if peak insulin, 2-h insulin, and total insulin values obtained by OGTT were greater than 150 (μU/mL), 75 (μU/mL), and 300 (μU/mL), respectively (12). For serum adiponectin and IL-6 levels, 8-10 mL of venous blood were taken from all patients. Following centrifugation at 3000 rpm for 10 min in sterile conditions, the serum samples were stored in clean and dry Eppendorf tubes in the freezer at -20 °C until analyzed. The ELISA method was used for measurements of plasma adiponectin (Ebioscience, Vienna, Austria reference no: BMS2032 lot no: 102162008) and IL-6 levels (Ebioscience, Vienna, Austria, reference no: BMS213/2, lot No: 101174060).

For about 24 hours, all patients underwent CGMS by means of a microdialytic system (Guardian^®^ REAL-Time CGMS and Sof-Sensor^®^, Medtronic MiniMed, Northridge, CA). In this system, a semipermeable microdialytic fiber is placed in the subcutaneous adipose tissue of the abdominal wall as a catheter guide. Hence, the membrane and interstitial space are in contact. Serum concentrations of glucose are roughly similar to those of the interstitial fluids. By using a biosensor based on the glucose-oxidase reaction, the glucose concentration in the device is measured. This device, which can be used in an outpatient basis, allows for the recording of daily routine activities. The registered data at the end of the test are downloaded and analyzed using software and are displayed in a final report showing the glycemic values recorded every 5 minutes. The mean 24-hour glycemia, its SD, and GV coefficient [GVC%=(SD/mean) x 100] were calculated for each CGMS test. The GVC % was assumed to represent GV.

### Statistical Analysis

Statistical analysis was performed using SPPS 21.0 (SPSS Inc., Chicago, IL, USA) software.

All data were given as mean ± SD values. The chi-square test was used to compare the frequency of the data. Homogeneity of the data was assessed using the Kolmogorov-Smirnov test. Differences in the means between the two groups were tested using the student’s t-test for data with normal distribution and the Mann-Whitney U test for data without normal distribution. Correlations were expressed by the Pearson’s or Spearman correlation coefficient according to data distribution. The results were expressed with a 95% confidence interval, and a p-value of less than 0.05 was considered statistically significant.

## RESULTS

A total of 50 patients (female/male: 31/19) with obesity and IR were included in the study. MS was diagnosed in 27 patients. The remaining patients had only IR without MS. Table 1 shows the clinical and laboratory characteristics of the groups. There was no difference between WC, BMI, total cholesterol, LDL-C, and adiponectin levels in the groups. Body FM, systolic and diastolic blood pressure, triglycerides, peak insulin in the OGTT, glycated hemoglobin (HbA1c), HOMA-IR, and IL-6 levels were significantly higher in the group with MS. WC, BMI, body fat FM, and triglyceride levels were negatively correlated with adiponectin level. Body weight, HbA1c, and triglyceride levels were positively correlated with IL-6. The results of the OGTT and 24-h continuous glucose monitoring in the patients with or without MS are shown in Table 2. GV was not different among the groups. Fasting insulin, 2-h insulin, peak insulin, total insulin, 2-h glucose levels in the OGTT, and HOMA-IR were positively correlated with GV, while no significant relation was detected with adiponectin and IL-6 levels (Table 3). Impaired fasting glucose and impaired glucose tolerance were detected in 7 and 6 patients, respectively when OGTT results were evaluated. Serum glucose levels during the day were found to exceed 200 mg/dL in 2/7 of patients with impaired fasting glucose and 1/6 of patients with impaired glucose tolerance according to CGMS data. The HbA1c values of these three patients were not different from the average (4.9%, 5.4%, and 5.7%).

## DISCUSSION

Adipokines secreted by adipose tissue and some cytokines are known to influence insulin sensitivity directly or indirectly by modulating insulin signal, glucose and lipid metabolism (4). In the literature, there are studies investigating the role of cytokines in obesity, diabetes, and MS and analyzing the efficacy of anti-inflammatory and inflammatory markers on endothelial dysfunction. Makni et al (5) reported that IL-6 levels are greater in children with MS than in children with IR only. Another study has found lower adiponectin levels in children diagnosed with MS compared with obese children without MS. In our study, IL-6 levels were significantly higher in the MS group compared to the other group. Although adiponectin levels were lower in the MS group, this difference was not statistically significant. These findings support the hypothesis that inflammation plays a role in the development of MS. Garanty-Bogacka et al (13) reported a significant reduction in IL-6 levels in obese children after an average weight reduction of 5.3 kg with a low-calorie diet for 6 months. IL-6 levels were significantly higher in the high HbA1c group than the low HbA1c group in a study in which type 2 DM diagnosed cases were divided into two groups according to HbA1c levels (14). We found a positive correlation between IL-6 levels and HbA1c in our study. These findings suggest that inflammation plays an important role in the increase in glycosylated hemoglobin levels and in the development of MS complications such as atherosclerosis, hypertension, and type 2 DM. In our study, a negative correlation was found between adiponectin levels and the parameters of obesity (waist circumference, BMI, FM) which were consistent with other studies (15,16). This result supports the studies which has reported that adiponectin strongly associates with obesity.

GV has been reported to contribute to the development of subclinical atherosclerosis, which leads to endothelial dysfunction by inducing oxidative damage (3). It is believed that oxidative damage occurs due to the excessive amounts of reactive oxygen species caused by fluctuations in blood glucose. Recent studies have shown that fluctuations in blood glucose levels have a worse effect on endothelial function compared to chronic hyperglycemia in both diabetic and non-diabetic individuals (2,3). There are studies in the literature investigating the relationship of GV with endothelial dysfunction and diabetes complications, but these studies are usually conducted in adult patients. There are no reports in the literature investigating the relationship of GV with MS and inflammatory markers in childhood obesity. Buscemi et al (3) reported that GV was higher in patients with type 2 DM compared to other obese individuals without diabetes and there was no difference found between adults with or without MS. In the same study, GV was found to positively correlate with WC, BMI, fasting insulin levels, HOMA-IR, and IL-6 levels. We did not find significant differences between the groups in terms of GV, however, GV positively correlated with BMI, IR parameters (HOMA-IR, fasting insulin, total and peak insulin levels in OGTT), and 2-h glucose in our study. Despite this positive association between GV and IR parameters, we did not find any significant relationship between GV and serum lipids and/or blood pressure. These results suggest that IR increases the fluctuations in blood glucose levels during the day. However, there is no evidence that dyslipidemia or hypertension increases GV in MS patients.

There is a limitation of this study that needs to be addressed. We did not have a healthy control group or a group of obese patients without IR in this study. If the MS group could be compared with controls, a significant difference in GV might have been detected.

In conclusion, this study drew attention to three important points. GV is not different among obese adolescents with IR and MS. There is a significant association between GV and IR parameters. However, other diagnostic criteria of MS (hypertension and/or dyslipidemia) or inflammation (elevated IL-6 levels) does not cause further increase in GV.

## Figures and Tables

**Table 1:**
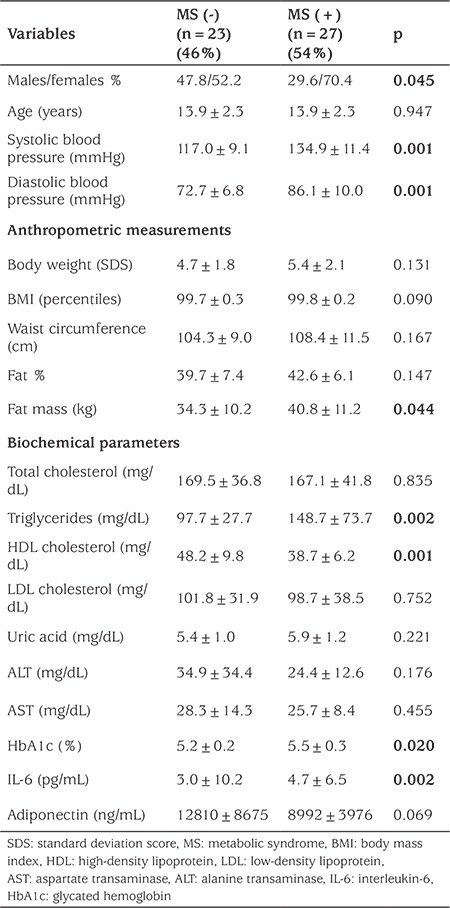
Clinical and laboratory characteristics of patients with or without metabolic syndrome

**Table 2:**
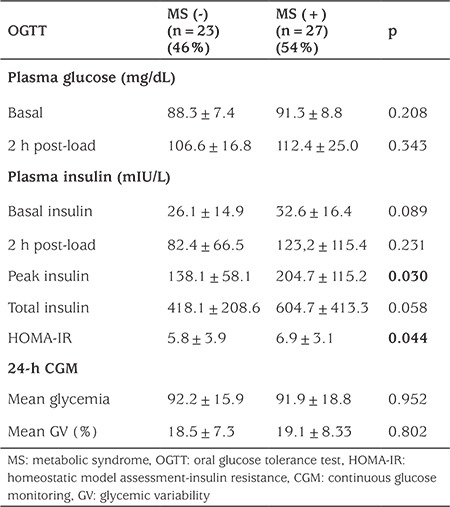
Oral glucose tolerance test and 24-h continuous glucose monitoring in patients with or without metabolic syndrome

**Table 3:**
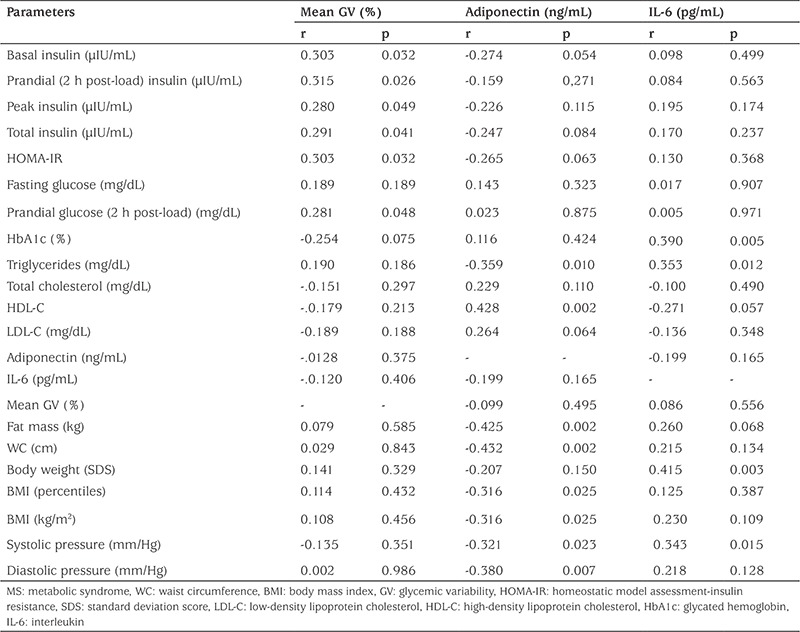
Correlations of clinical and laboratory variables with adiponectin, interleukin-6, and mean glycemic variability
